# Modified Martin Procedure for Megacystis Microcolon Intestinal Hypoperistalsis Syndrome (MMIHS)

**DOI:** 10.1007/s12098-024-05404-7

**Published:** 2025-02-14

**Authors:** Li Tian, Chensen Ma, Peng Cao, Donghai Yu

**Affiliations:** 1 Department of Pediatric Surgery, Affiliated Tongji Hospital, Tongji Medical College, Huazhong University of Science and Technology, Wuhan, 430030 Hubei China; 2 Hubei Clinical Center of Hirschsprung’s Disease and Allied Disorders, Wuhan, 430030 Hubei China

**Keywords:** MMIHS, Martin, Bishop–koop, Clinical outcome

## Abstract

**Objectives:**

To evaluate the therapeutic effect of the modified Martin procedure for patients with megacystis microcolon intestinal hypoperistalsis syndrome (MMIHS).

**Methods:**

The authors retrospectively analyzed the medical records of patients diagnosed with MMIHS who underwent surgical treatment at their institute between August 2019 and June 2023. The modified Martin procedure and Bishop–Koop procedure were performed for the patients. Data, including demographics, operative time, intraoperative blood loss, complications and clinical outcomes, were collected.

**Results:**

A total of 11 children with MMIHS were enrolled in this study. The mean age of the patients was 4.08 ± 1.69 y, and seven cases (63.64%) were male. The average operation time was 312.09 ± 108.88 min, with an average intraoperative blood loss of 25.82 ± 6.42 ml. The first postoperative defecation time was 3.60 ± 0.91 d. Patients took an average of 4.10 ± 0.40 d to resume eating after surgery, and their average length of hospital stay was 24.64 ± 4.52 d. Postoperative follow-up results showed that of the nine surviving children, two patients required intermittent intravenous nutrition support, and seven patients had successfully transitioned from parenteral nutrition support. In this study, two patients eventually died of multiple organ failure.

**Conclusions:**

The modified Martin procedure is a promising method for treating MMIHS. The patients exhibited positive nutritional status and satisfactory outcomes during the follow-up period.

**Supplementary Information:**

The online version contains supplementary material available at 10.1007/s12098-024-05404-7.

## Introduction

Megacystis microcolon intestinal hypoperistalsis syndrome (MMIHS) is a rare congenital condition that causes functional intestinal obstruction in newborns, often leading to mortality. This syndrome was initially described by Berdon in 1976 and is characterized by a distended abdomen caused by a non-obstructed dilated bladder, microcolon, and a decrease and/or absence of intestinal peristalsis [1]. It is a type of chronic intestinal pseudo-obstructive disease characterized by intestinal agenesis, however, unlike the more common Hirschsprung disease, ganglion cells are present in rectal biopsies. Most patients require parenteral nutrition (PN) because of gastrointestinal dysfunction that makes oral feeding difficult, which can lead to life-threatening complications such as parenteral nutrition associated cholestasis and catheter-related bloodstream infections [2]. It has been reported that most patients with MMIHS die from complications related to gastrointestinal motility disorders [3].

In recent years, several case reports and experimental studies have been published to describe the clinical features, diagnosis, and treatment options for this syndrome. However, the precise cause and treatment of the disease are still a matter of controversy. Particularly, in terms of surgical treatment for MMIHS, there is currently no established method or standard. In this study, the authors retrospectively analyzed the clinical data of 11 children with MMIHS who underwent surgical treatment with the modified Martin procedure. The findings suggest that surgery may be necessary to improve the prognosis of patients with MMIHS.

## Material and Methods

The authors retrospectively analyzed the medical records of 11 patients diagnosed with MMIHS who underwent surgical treatment at their institute between August 2019 and June 2023. Prior to surgery, all patients underwent preoperative barium enema, recto-anal manometry, and rectal mucosal biopsy. Additionally, the colon was irrigated daily with saline until the day of surgery. Intermittent indwelling catheterization was performed according to the condition before surgery to prevent bladder overexpansion. Nutritional support was provided before surgery. The following information was recorded: age, gender, height, weight, operation time, blood loss, length of hospital stay, laboratory test results, postoperative complications and clinical outcomes. The follow-up period was one year. This study was approved by the Ethics Committee of Tongji Hospital, Tongji Medical College, Huazhong University of Science and Technology (TJ-IRB202407074). Informed consent was obtained from all patients’ parents before all diagnostic procedures and surgery according to the 1964 Helsinki Declaration principles.

Modified Martin procedure: After the induction of general anesthesia, three 5 mm trocars were inserted on both sides of the umbilical ring and on the left lateral abdomen. The mesocolon was first released from the ascending colon up to the upper part of the sigmoid colon. Endoscopic linear cutters were used to separate the terminal ileum. The ascending colon, transverse colon, and descending colon were then resected. Finally, the terminal ileum and 6–8 cm distal sigmoid colon were anastomosed using endoscopic linear cutters and placed side by side. In addition, a transrectal tube was inserted into the intestine above the anastomosis site.

**Fig. 1:**
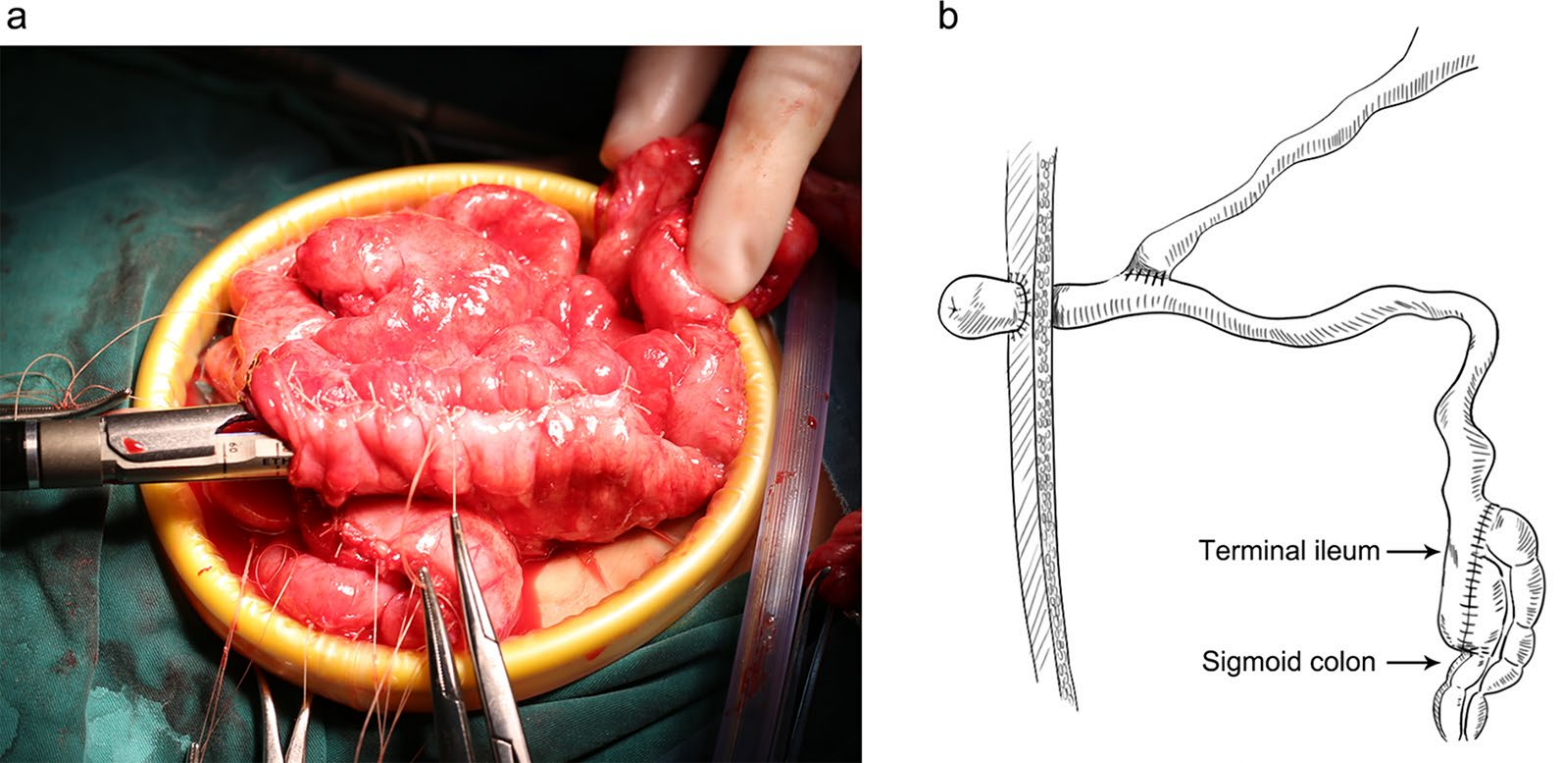
**(a)** Intraoperative picture of terminal ileum and sigmoid colon being anastomosed using endoscopic linear cutters. **(b)** Schematic representation of the Modified Martin procedure

Bishop–Koop procedure: The intestine was transected approximately 30 cm above the anastomosis. The proximal and distal segments of the intestine were then connected in an end-to-side manner, with exteriorization of the end of the distal segment as an end-stoma (Fig. 1). In addition, silicone tubes with side holes were placed into the proximal and distal segments of the intestines through the stoma to support, drain, and arrange the intestines.

Following surgery, all patients were administered intravenous antibiotics and other necessary treatment. They were kept on total parenteral nutrition, and once bowel sounds returned and stool was passed, a liquid diet was initiated, followed by a semi-liquid diet and oral enteral nutrition solution. The rectal tube and silicone arrangement tube were removed 5–7 d and 2 wk after surgery respectively. Parents were instructed on stoma care before discharge. Patients were discharged once they were able to tolerate total enteral nutrition and were in a stable clinical condition.

Measurement data are expressed as the mean ± standard deviation, and counting data are expressed as frequency (percentage). Descriptive statistics were used to describe patient demographics and clinical information. Quantitative data were analyzed using Student’s t test, while categorical data were analyzed using Pearson chi-square test or Fisher’s exact probability method. Statistical significance was set at *P* < 0.05. All statistical analyses were done using the IBM Statistical Package for Social Science (SPSS) version 22 (IBM Corp., Chicago).

## Results

Eleven children with MMIHS were enrolled in the study. The mean age of the patients was 4.08 ± 1.69 y, and seven patients (63.64%) were male. The primary clinical symptoms observed in these children included abdominal distension, vomiting, recurrent enteritis, and malnutrition. Additionally, most patients had previously undergone more than 2 surgeries in other hospitals, such as ostomy, intestinal biopsy, and intestinal adhesiolysis. In addition, the genetic testing results of these patients showed that nine cases had ACTG2 mutations, one case had MYLK mutation, and one child had a heterozygous deletion of chromosome 10 (Table 1).

**Table 1:** Clinical characteristics of study population (*N* = 11)

Characteristics	Value
Age, year, Mean ± SD	4.08 ± 1.69
Male, n (%)	7 (63.64)
Weight, Mean ± SD	10.63 ± 3.84
BMI, Mean ± SD	12.64 ± 2.03
Gene mutation, n (%)	
ACTG2	9 (81.82)
MYLK	1 (9.09)
Chromosome 10 deletion	1 (9.09)
Symptoms, n (%)	
Abdominal distension	10 (90.91)
Vomiting	3 (27.27)
Recurrent enteritis	5 (45.45)
Number of previous surgeries, n (%)	
1 ~ 2	2 (18.18)
3 ~ 4	8 (72.73)
≥ 5	1 (9.09)
Perioperative characteristics	
Operative time (min), Mean ± SD	312.09 ± 108.88
Intraoperative bleeding volume (ml), Mean ± SD	25.82 ± 6.42
First postoperative anal defecation time (days), Mean ± SD	3.60 ± 0.91
First postoperative feeding time (days), Mean ± SD	4.10 ± 0.40
Length of stay (days), Mean ± SD	24.64 ± 4.52
Short-term complications (n, %)	
Anastomotic bleeding	1 (9.09)
Incision infection	2 (18.18)
Enterocolitis	2 (18.18)
Intermittent abdominal distension	2 (18.18)
Perianal skin ulceration	4 (36.36)
Long-term outcome (n, %)	
Dependence on PN	
Intermittent	2 (18.18)
Independent	7 (63.64)
Survival	9 (81.82)
Dead	2 (18.18)

*BMI* Body mass index

Supplementary Fig. S1 presents the findings of the preoperative gastrointestinal barium contrast imaging in a representative patient. The intestine was extensively dilated and exhibited impaired emptying function. Moreover, reflexes of rectal and anal manometry were observed, and rectal biopsy confirmed the presence of ganglion cells.

In this study, the average operation time was 312.09 ± 108.88 min, with an average intraoperative blood loss of 25.82 ± 6.42 ml. The first postoperative defecation time was 3.60 ± 0.91 d. Patients took an average of 4.10 ± 0.40 d to resume eating after surgery, and their average length of hospital stay was 24.64 ± 4.52 d (Table 1). Supplementary Fig. S2 shows that the volume of discharge from the stoma gradually decreased.

Laboratory test results showed that 3 d after surgery, the levels of white blood cells (WBC), C-reactive protein (CRP), and procalcitonin (PCT) decreased significantly compared with those at 10 d after surgery. There was no significant difference in liver function among all patients within 10 d of surgery (Table 2).

**Table 2:** Laboratory test results after surgery (*N* = 11)

Variables	3 d after surgery	10 d after surgery	*P*
WBC (10^^9^), Mean ± SD	14.57 ± 1.75	8.32 ± 1.89	< 0.001
Hb (g/L), Mean ± SD	111.09 ± 9.95	120.82 ± 8.98	0.061
CRP (mg/L), Mean ± SD	35.45 ± 18.05	3.13 ± 4.15	< 0.001
PCT (ng/mL), Mean ± SD	3.59 ± 2.16	0.27 ± 0.33	0.001
ALT (U/L), Mean ± SD	33.82 ± 25.00	42.19 ± 16.42	0.111
AST (U/L), Mean ± SD	36.54 ± 23.98	44.73 ± 17.15	0.050
ALB (g/L), Mean ± SD	40.67 ± 1.90	42.24 ± 2.87	0.159

*ALB* Albumin; *ALT* Alanine aminotransferase; *AST* Aspartate aminotransferase; *CRP* C-reactive protein; *Hb* Hemoglobin; *PCT* Procalcitonin; *WBC* White blood cells

Postoperative complications included anastomotic bleeding (1/11), incision infection (2/11), enterocolitis (2/11), intermittent abdominal distension (2/11), and perianal skin ulceration (4/11) (Table 1). Pathological examination revealed ganglion cells in the submucosal and intermuscular regions of the colon wall. However, these cells were observed to be dysplastic and their number decreased.

The results of postoperative follow-up revealed that 2 patients required intermittent intravenous nutrition support, and 7 patients had successfully transitioned from parenteral nutrition support. Supplementary Fig. S3 shows that patients tended to gain weight after surgery, which reflected the improvement of their nutritional status. In this study, two patients eventually died of multiple organ failure at 3 and 7 mo due to long-term dependence on parenteral nutrition and recurrent abdominal distension and sepsis after surgery (Table 1).

## Discussion

Previous studies have revealed that MMIHS is a highly distressing disease with an average life expectancy of less than six months [4]. However, there is a glimmer of hope, as a small number of patients have managed to survive into adulthood with the aid of professional medical intervention and care. Furthermore, it is encouraging to note that overall mortality rates have shown significant improvement over the last four decades [5]. Although there is currently no universally accepted treatment protocol for MMIHS, authors’ preliminary clinical practice and review of relevant literature from the past decade indicate that surgical intervention can effectively prolong the survival expectancy of children and enhance their clinical prognosis (Supplementary Table S1) [2, 3, 6–14].

There have been reports indicating a significant correlation between MMIHS and gene mutations, specifically mutations in ACTG2, MYL9, MYH11, MYLK, and LMOD1 [15, 16]. These gene mutations have been found to cause a reduction in filamentous actin, elongation of dense cytoskeletal bodies, and impairment of intestinal smooth muscle contractility. It suggests that there is a relationship between abnormal smooth muscle cytoskeleton-contraction coupling and the development of MMIHS.

For patients with MMIHS, early exploratory laparotomy is necessary to decompress the severely dilated intestine and rule out congenital anomalies, such as intestinal atresia and Hirschsprung’s disease. However, all patients included in this study underwent more than two surgeries in other hospitals, including enterostomy, re-enterostomy after stoma closure, and intestinal adhesiolysis. This suggests that some pediatric surgeons may lack a sufficient understanding of this disease or that their treatment methods are suboptimal. Previous literature classified MMIHS as a “variant of Hirschsprung disease”. Friedmacher and Puri found that 77% of children with MMIHS had normal ganglion cells in the submucosal colon and myenteric plexus, but some children also had reduced/increased ganglion cells or immature ganglion cells [17]. In this study, pathological results showed abnormal development and reduced number of intestinal ganglion cells in patients with MMIHS. Although the number of cases is relatively small, the results are consistent with previous studies.

Martin surgery, a side-to-side anastomosis of the ileum-descending colon, is considered a suitable procedure for total colonic aganglionosis [18]. Patients with MMIHS face two major challenges: dynamic intestinal obstruction caused by intestinal motility disorders and adhesive intestinal obstruction aggravated by multiple surgeries. In this study, the modified Martin procedure and Bishop-Koop ostomy were performed to address the above two problems. The advantages of these surgical improvements are as follows: (1) removal of most of the dysfunctional colon while retaining the distal sigmoid colon for water absorption and prevention of intractable diarrhea; (2) side-to-side anastomosis reduces the risk of anastomotic stenosis and allows for the formation of a “pouch” to prevent fecal incontinence; (3) Bishop’s stoma in the proximal small intestine helps reduce intestinal pressure and prevent abdominal distension or intestinal obstruction; (4) it can not only relieve the symptoms of dynamic intestinal obstruction, but also prevent adhesive intestinal obstruction. Of the total participants, nine children successfully survived and achieved partial or complete independence from intravenous nutrition after surgery.

It is important to acknowledge the limitations of this study. First, the sample size was relatively small, which can be attributed to the rarity of MMIHS. Second, this study focused solely on improving intestinal dysfunction and did not address the treatment of the urinary system. It is worth noting that three children in the study required intermittent indwelling urinary catheters. Finally, it is crucial for future research to observe the long-term outcomes of patients.

## Conclusions

The treatment of MMIHS is challenging and lacks a standardized approach. However, present study indicated that the modified Martin procedure is a promising method for treating MMIHS. The patients exhibited positive nutritional status and satisfactory outcomes during the follow-up period. Nevertheless, further extensive studies are necessary to compare the modified Martin and other procedures.

## Electronic Supplementary Material

Below is the link to the electronic supplementary material.

Supplementary Material 1

Supplementary Material 2

Supplementary Material 3

Supplementary Material 4
