# Supplementary material for: Modified Martin Procedure for Megacystis Microcolon Intestinal Hypoperistalsis Syndrome (MMIHS)
Source: Indian J Pediatr. 2025 Feb 14;92(12):1315–9. doi: 10.1007/s12098-024-05404-7 (PMC12647210; doi:10.1007/s12098-024-05404-7)
Supplement: Supplementary file 1 — Supplementary Material 1 [file 12098_2024_5404_MOESM1_ESM.docx]

**Supplementary Table S1** Surgical treatments of MMIHS in the past decade

| **Authors** | **Nation** | **Year** | **Surgical treatment** | **Number** | **Alive** | **Follow-up (month)** |
| --- | --- | --- | --- | --- | --- | --- |
| Jain et al. [6] | India | 2011 | Ileostomy | 1 | 0 | / |
| Ravindra et al. [7] | United States | 2012 | Multivisceral transplantation | 1 | 1 | 16 |
| Huang et al. [8] | China (Taiwan) | 2012 | Isolated intestinal transplantation | 1 | 1 | 48 |
| López-Muñoz et al. [3] | Mexico | 2012 | Intestinal partial resection | 1 | 1 | 60 |
| Mc Laughlin and Puri [9] | Ireland | 2013 | Bowel transplant | 1 | 1 | 24 |
| Hiradfar et al. [10] | Iran | 2013 | Ileostomy | 1 | 0 | / |
| Liaqat et al. [11] | Pakistan | 2015 | Ileostomy | 1 | 1 | 1 |
| Soh et al. [12] | Japan | 2015 | Jejunostomy | 11 | 4 | / |
|  |  |  | Ileostomy | 1 | 1 | / |
|  |  |  | Colostomy | 5 | 4 | / |
|  |  |  | Resection | 4 | 2 | / |
| Xiao and Chen [13] | China | 2015 | Colectomy | 2 | 1 | 12 |
|  |  |  | Soave | 2 | 1 | 12 |
|  |  |  | Ladd’s | 1 | 1 | 12 |
| Wymer et al. [2] | United States | 2016 | Ileostomy | 2 | 2 | 12 |
|  |  |  | Colectomy, ileostomy | 1 | 1 | >12 |
|  |  |  | Completion colectomy | 1 | 1 | 12 |
| Tao et al. [14] | China | 2016 | Ileostomy | 1 | 1 | 8 |
|  |  |  | Bishop-Koop jejunostomy | 1 | 1 | 12 |
