# Supplementary material for: Modified Martin Procedure for Megacystis Microcolon Intestinal Hypoperistalsis Syndrome (MMIHS)
Source: Indian J Pediatr. 2025 Feb 14;92(12):1315–9. doi: 10.1007/s12098-024-05404-7 (PMC12647210; doi:10.1007/s12098-024-05404-7)
Supplement: Supplementary file 2 — Supplementary Material 2 [file 12098_2024_5404_MOESM2_ESM.docx]

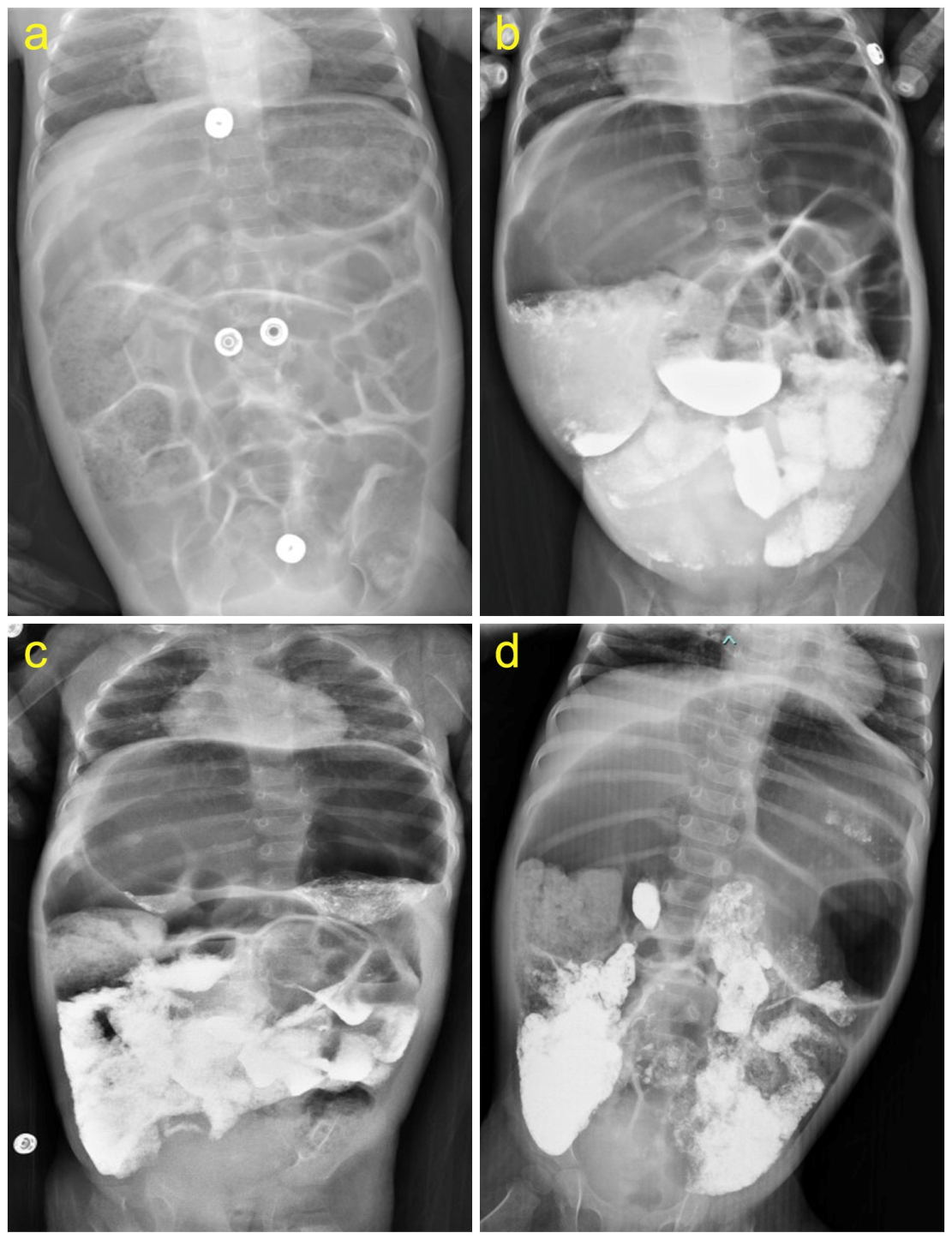


**Supplementary Fig. S1** Preoperative gastrointestinal barium contrast imaging of a

representative patient. **a**, **b**, **c** and **d** represent X-rays taken at 0, 6, 12, and 24 h after

administration of contrast agent, respectively.
