# Supplementary figures and images for: Modified Martin Procedure for Megacystis Microcolon Intestinal Hypoperistalsis Syndrome (MMIHS)
Source: Indian J Pediatr. 2025 Feb 14;92(12):1315–9. doi: 10.1007/s12098-024-05404-7 (PMC12647210; doi:10.1007/s12098-024-05404-7)

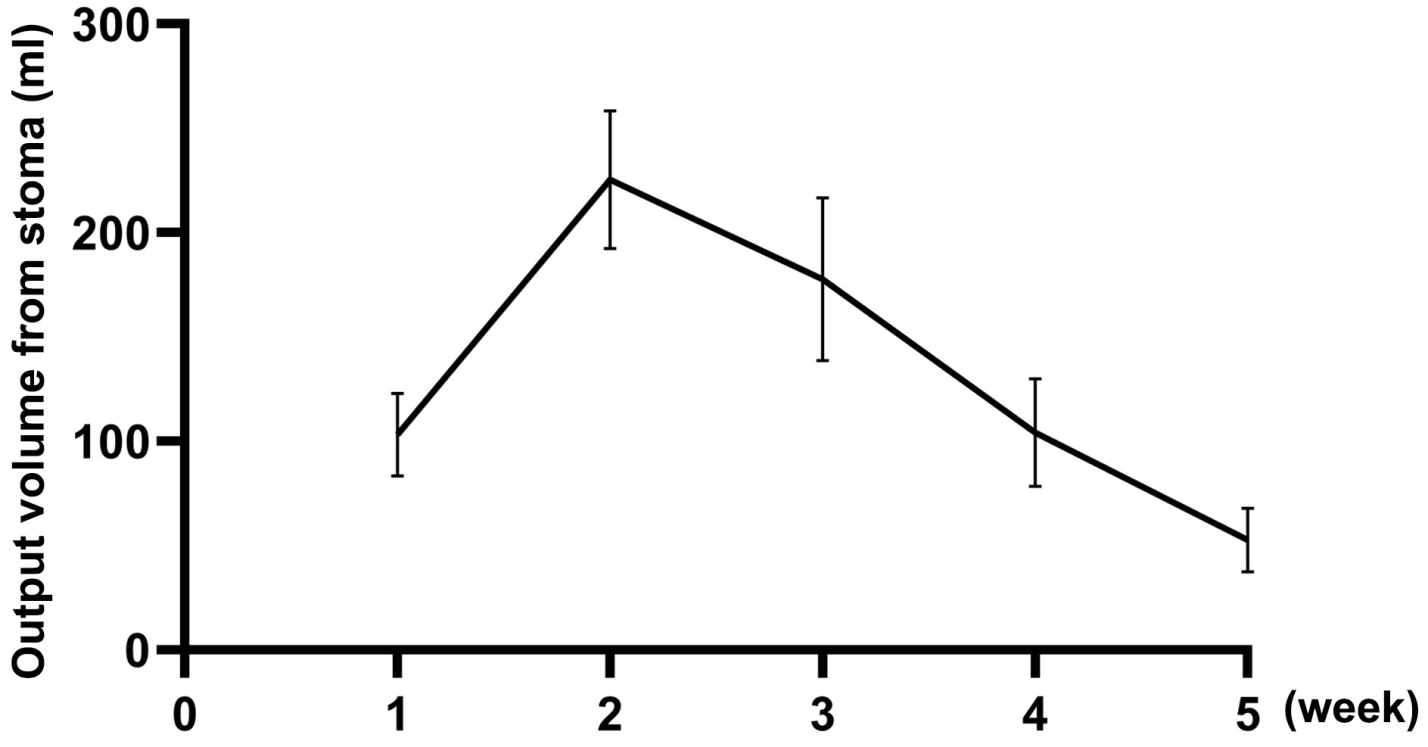


**Supplementary Fig. S2** The volume of discharge from the stoma at different times

after surgery.

Supplement: Supplementary file 3 — Supplementary Material 3 [file 12098_2024_5404_MOESM3_ESM.docx]

**
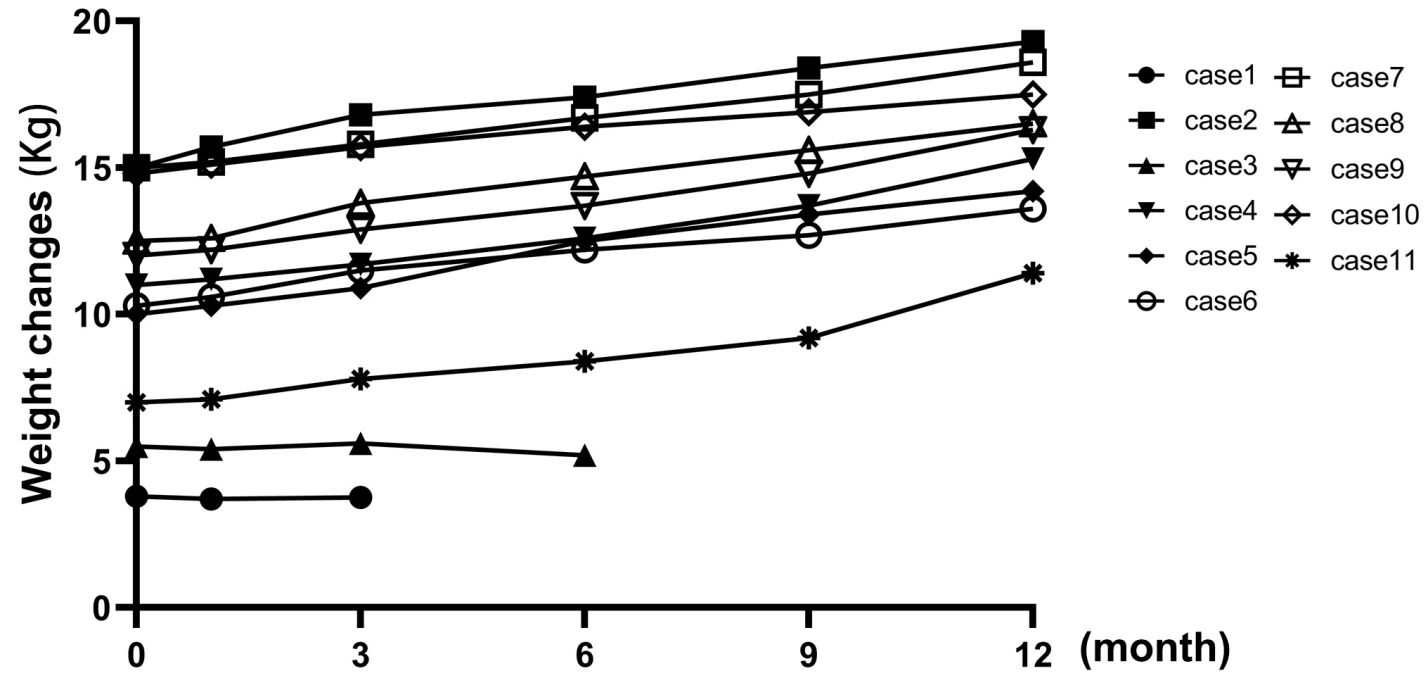
**

**Supplementary Fig. S3** Weight changes in children with MMIHS after surgery.

Supplement: Supplementary file 4 — Supplementary Material 4 [file 12098_2024_5404_MOESM4_ESM.docx]
